# Muscle quality index is associated with advanced stages in patients with cardiovascular-kidney-metabolic syndrome: A cross-sectional study

**DOI:** 10.1097/MD.0000000000049366

**Published:** 2026-06-19

**Authors:** Huang Yu

**Affiliations:** a Department of Cardiovascular Medicine, Xinfeng County People’s Hospital, Ganzhou, Jiangxi, China.

**Keywords:** cardiovascular-kidney-metabolic syndrome, cross-sectional study, muscle quality index, NHANES, sex differences, threshold effect

## Abstract

Cardiovascular-kidney-metabolic (CKM) syndrome represents a complex systemic condition involving interconnected dysfunctions across metabolic, renal, and cardiovascular systems, presenting significant prevalence rates and imposing considerable clinical and economic challenges. Muscle mass and handgrip strength are recognized as independent prognostic indicators in cardiovascular, kidney, and metabolic diseases. The muscle quality index (MQI), integrating measures of muscle strength and mass, has demonstrated relevance for various health outcomes, but its association with advanced CKM syndrome remains unclear. This investigation employed a cross-sectional design utilizing National Health and Nutrition Examination Survey data from 2011 to 2014. The MQI was calculated as the ratio of handgrip strength to appendicular skeletal muscle mass. Advanced CKM syndrome was defined as stages 3 to 4 according to American Heart Association criteria. We employed multivariable logistic regression, restricted cubic spline analysis, and stratified analyses to examine the association between MQI and advanced CKM syndrome. Among 2012 participants, 4.7% had advanced CKM syndrome. Elevated MQI was associated with a reduced prevalence of advanced CKM syndrome (odds ratio = 0.54 per unit increase; 95% confidence interval = 0.29–0.99), with a threshold effect observed at MQI = 2.66, indicating stronger protection below this level. Notably, significant effect modifications by sex (*P*-interaction = .0083) and income (*P*-interaction < .0001) were observed: the inverse association was evident only in males and the highest-income individuals. Muscle quality exhibits a threshold-dependent effect against advanced CKM syndrome, with enhanced benefits in males and high-income populations. This suggests that maintaining muscle quality may be an important clinical consideration. Prospective longitudinal studies are warranted to clarify this association.

## 1. Introduction

Cardiovascular-kidney-metabolic (CKM) syndrome represents a multisystem disorder involving interconnected dysfunctions across metabolic, renal, and cardiovascular systems, leading to multiorgan damage and adverse clinical outcomes.^[1]^ Epidemiological data indicate that up to 89.5% of US adults meet criteria for CKM stages 1 to 4, with 14.6% progressing to advanced stages (stages 3–4) associated with established cardiovascular disease (CVD).^[2,3]^ These advanced stages are linked to substantial healthcare expenditures, primarily due to the CVD burden.^[1]^ The growing prevalence and economic impact of CKM syndrome underscore the importance of identifying effective strategies for its management and prevention.

Reduced muscle mass and diminished handgrip strength (HGS) are independently associated with adverse outcomes across cardiovascular, kidney, and metabolic diseases. For cardiovascular outcomes, meta-analyses demonstrate that lower HGS significantly increases the risk of CVDs (hazard ratio [HR] = 1.63, 95% confidence interval [CI] = 1.36–1.96) and all-cause mortality (HR = 1.41, 95% CI = 1.30–1.52).^[4]^ Dose–response analyses confirm these associations extend to cancer and cardiovascular mortality.^[5]^ Reduced HGS independently predicts adverse outcomes in heart failure,^[6]^ while sarcopenia (possible or confirmed) elevates CVD risk (HR = 1.22–1.33)^[7]^ and accelerates disease progression in older adults.^[8]^

In metabolic disorders, lower HGS is associated with increased metabolic syndrome risk (odds ratio [OR] = 2.59, 95% CI = 2.06–3.25)^[9]^ and insulin resistance across different age groups,^[10]^ and displays inverse relationships with metabolic syndrome components.^[11]^ Combined low muscle mass and high fat mass further increase metabolic syndrome risk (incidence rate ratio = 1.90, 95% CI = 1.44–2.50),^[12]^ as does an elevated fat-to-muscle ratio.^[13]^ Declining muscle mass has also been identified as a predictor of increased diabetes risk.^[14]^

Within kidney disease populations, meta-analyses show that lower HGS predicts higher mortality risk in dialysis patients (relative risk = 1.88, 95% CI = 1.51–2.33),^[15]^ with dose–response thresholds predictive of all-cause mortality in chronic kidney disease (CKD).^[16]^ Sarcopenia further increases mortality risk among dialysis patients (HR = 1.87, 95% CI = 1.35–2.59),^[17]^ and its coexistence with CKD is associated with a marked rise in all-cause mortality (HR = 2.59, 95% CI = 2.17–3.09).^[18]^ Overall, these findings establish muscle strength and mass as important prognostic indicators across the CKM spectrum.

Although these studies consistently highlight the independent prognostic value of HGS and muscle mass, most have evaluated these parameters separately rather than as composite measures. The muscle quality index (MQI), defined as the ratio of HGS to appendicular skeletal muscle mass (ASM),^[19,20]^ offers a comprehensive evaluation of muscle health and function. Recent evidence links lower MQI to increased risks of depression,^[19]^ arthritis,^[21]^ urinary incontinence,^[22]^ diabetes,^[23]^ and hyperuricemia,^[24,25]^ suggesting its added value in metabolic health assessment. However, research examining the association between MQI and advanced CKM syndrome is scarce.

Therefore, using nationally representative National Health and Nutrition Examination Survey (NHANES) data, this study investigated the relationship between MQI and advanced CKM syndrome. We hypothesized that elevated MQI would be inversely associated with advanced CKM syndrome prevalence. In addition, we explored potential nonlinear relationships and possible variations in this association across population subgroups. This investigation aims to provide epidemiological evidence regarding the association between muscle quality and advanced CKM syndrome.

## 2. Materials and methods

### 2.1. Survey description and population

This analysis utilized data from the NHANES, a nationwide health surveillance program administered by the National Center for Health Statistics. The survey employs multistage probability sampling with stratification and clustering to represent the civilian, noninstitutionalized United States population. All participants provided written informed consent when recruited, and the National Center for Health Statistics Ethics Review Board approved the study methodology. NHANES data were accessed for research purposes on March 2, 2025. Since this study involved secondary analysis of publicly available, de-identified NHANES data, no external ethical approval was required for this secondary data analysis.

**Figure 1.**
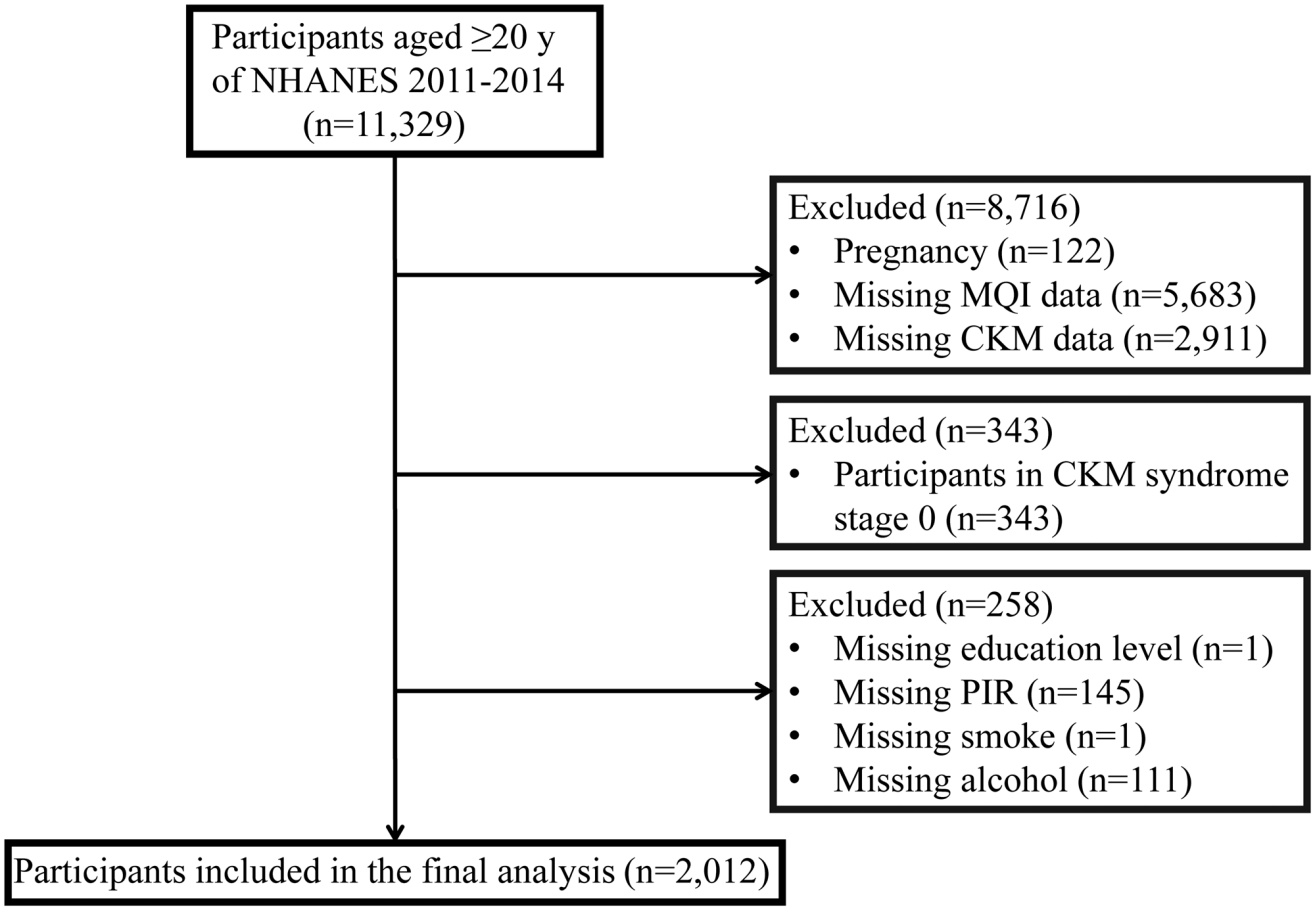
Flowchart of study participant selection. CKM = cardiovascular-kidney-metabolic, MQI = muscle quality index, NHANES = National Health and Nutrition Examination Survey, PIR = poverty-to-income ratio.

We utilized data from NHANES 2011 to 2014 cycles, which are the only cycles containing grip strength measurements necessary for calculating the MQI. Among 11,329 adults aged ≥20 years, we applied the following exclusion criteria: pregnant individuals (n = 122) to avoid potential physiological confounding; participants with missing data on MQI (n = 5683) or CKM syndrome (n = 2911); those with incomplete covariate data (education, n = 1; income level, n = 145; smoking, n = 1; alcohol consumption, n = 111); and individuals with CKM syndrome Stage 0 (n = 343), as Stage 0 represents absence of CKM syndrome according to established clinical classifications.^[2]^ The final analytic sample consisted of 2012 participants with complete data (Fig. 1).

### 2.2. Muscle quality index

MQI was calculated as the ratio of HGS to ASM (kg/kg).^[26]^ HGS was assessed using a calibrated hand dynamometer, with participants standing and performing 3 trials for each hand at 60-second intervals. The maximum value for each hand was recorded, and the sum of these values was used as the final HGS. Participants were eligible for measurement if they had not undergone surgery within the past 3 months and reported no hand or wrist pain. ASM was evaluated using dual-energy X-ray absorptiometry and represented the total lean tissue mass from both arms and legs, excluding fat and bone mineral components.^[27,28]^ Following the approach of Lopes et al,^[26]^ MQI was classified into normal, low, and extremely low groups based on sex-specific cutoffs at 1 and 2 standard deviations below the mean, respectively (Table S1, Supplemental Digital Content 1).

### 2.3. Definition of CKM syndrome

CKM syndrome staging was determined according to the American Heart Association framework,^[29]^ referencing the methodology applied to NHANES data by Aggarwal et al.^[2]^ This classification consists of 5 stages: Stage 0 indicates the absence of cardiovascular, kidney, or metabolic abnormalities, defined by normal body mass index (BMI), waist circumference, glucose metabolism, lipid profiles, and blood pressure. Stage 1 reflects excess or dysfunctional adipose tissue, identified by elevated BMI, increased waist circumference, or prediabetes. Stage 2 is characterized by metabolic abnormalities including hypertension, diabetes, dyslipidemia, metabolic syndrome, or moderate-to-high CKD risk. Stage 3 encompasses very-high CKD risk or high 10-year CVD risk. Stage 4 is defined by CVD. Detailed diagnostic criteria for each stage are presented in Table S2, Supplemental Digital Content 2. CKM syndrome was defined as Stage 1 or higher, while advanced stages were considered as Stage 3 and Stage 4.^[2]^

### 2.4. Covariates

Based on prior literature,^[30]^ we selected demographic and lifestyle factors as covariates, including age, sex, race/ethnicity, education, marital status, family income measured by poverty-to-income ratio (PIR), smoking, and alcohol status. Detailed classifications are presented in Table S3, Supplemental Digital Content 3.

Physiological and biochemical variables commonly used as covariates in similar studies, including blood pressure, blood glucose, lipid profile, BMI, renal function parameters, and history of CVD, were not included in our regression models, as these parameters are already incorporated in the diagnosis and staging of CKM syndrome.^[2,29]^ Including these variables would introduce multicollinearity into the analyses, potentially distorting the effect estimates. We assessed for multicollinearity among selected covariates using variance inflation factor analysis, confirming that all variance inflation factor values remained below 5 (refer to Table S4, Supplemental Digital Content 4).

### 2.5. Statistical analysis

Statistical analyses incorporated survey weights to ensure population-representative estimates. Descriptive statistics are presented as mean ± SE for continuous variables and weighted proportion with SE for categorical variables. Between-group differences were evaluated using weighted *t* tests for continuous variables and weighted chi-square tests for categorical variables. The association between MQI and advanced CKM syndrome was evaluated using multiple logistic regression analyses. We conducted 3 analytical models: Model 1 (no covariates were adjusted), Model 2 (adjusted for age, sex, and race), and Model 3 (further adjusted for educational level, marital status, family income, smoking, and alcohol status). We assessed nonlinear associations via 4-knot restricted cubic splines and applied segmented regression to detect breakpoints, with optimal breakpoints determined through log-likelihood ratio tests and recursive model fitting. In addition, subgroup analyses were performed across demographic, socioeconomic, and lifestyle factors. Effect modification was assessed using likelihood ratio tests for interaction. Statistical analyses were performed using R software (version 4.4.2; R Core Team, R Foundation for Statistical Computing, Vienna, Austria, https://www.R-project.org/). Two-sided hypothesis testing was employed throughout, with statistical significance defined as *P* < .05.

## 3. Results

### 3.1. Baseline characteristics

The study included 2012 participants with CKM syndrome, representing a weighted population of 47,301,021 individuals. Among them, 98 participants (4.7%) were classified as having advanced CKM syndrome. Patients with advanced CKM stages exhibited a lower mean MQI compared to those with non-advanced stages (3.12 ± 0.11 vs 3.35 ± 0.02, *P* = .0338). MQI distribution differed significantly between groups (*P* = .0048), with a greater proportion of individuals in the advanced stage exhibiting extremely low MQI (43.25 ± 5.40% vs 29.11 ± 1.48%). Only 33.84 ± 5.09% of participants with advanced CKM had normal MQI, compared to 52.54 ± 1.43% in the non-advanced group. In addition, individuals with advanced CKM were older, had lower educational levels and family income, and demonstrated higher smoking prevalence compared with earlier-stage participants (Table 1).

**Table 1:** Weighted baseline characteristics of study population with CKM syndrome.

Characteristics	Patients with CKM syndrome			*P* value
	Total (N = 47,301,021, n = 2012)	Non-advanced stages (N = 45,094,269, n = 1914)	Advanced stages (N = 2,206,752, n = 98)	
Age, yr, mean ± SE	40.36 ± 0.42	39.89 ± 0.40	49.87 ± 0.84	<.0001
Sex, % (SE)				.4980
Male	54.67 (1.55)	54.46 (1.51)	58.84 (6.61)	
Female	45.33 (1.55)	45.54 (1.51)	41.16 (6.61)	
Race/ethnicity, % (SE)				.3936
Mexican American	9.64 (1.32)	9.81 (1.33)	6.33 (2.08)	
Other Hispanic	6.06 (1.15)	5.89 (1.08)	9.55 (3.85)	
Non-Hispanic White	66.02 (2.68)	66.12 (2.63)	64.00 (8.51)	
Non-Hispanic Black	11.18 (1.28)	11.03 (1.26)	14.32 (3.73)	
Other race	7.09 (0.72)	7.15 (0.72)	5.80 (2.82)	
Education level, % (SE)				.0437
<High school	14.75 (1.48)	14.31 (1.52)	23.60 (5.59)	
High school or equivalent	21.74 (1.94)	21.20 (1.80)	32.70 (10.20)	
>High school	63.52 (2.64)	64.49 (2.67)	43.70 (7.48)	
Marital status, % (SE)				.0723
Married/living with a partner	61.78 (1.94)	62.26 (1.93)	51.97 (9.25)	
Divorced/separated/widowed	14.42 (1.25)	13.82 (1.22)	26.64 (6.11)	
Never married	23.80 (1.77)	23.92 (1.70)	21.39 (6.91)	
Poverty-to-income ratio, % (SE)				.0009
<1.3	26.01 (2.56)	25.22 (2.47)	42.06 (6.66)	
1.3–3.5	34.28 (1.72)	34.32 (1.65)	33.53 (5.81)	
≥3.5	39.71 (2.70)	40.46 (2.66)	24.41 (5.45)	
Smoking status, % (SE)				<.0001
Never	56.73 (1.85)	58.05 (1.70)	29.85 (5.12)	
Former	19.57 (1.27)	19.63 (1.32)	18.33 (4.95)	
Now	23.70 (1.94)	22.32 (1.82)	51.82 (5.31)	
Alcohol status, % (SE)				.6356
Never	8.55 (1.18)	8.49 (1.16)	9.75 (3.84)	
Low to moderate	63.23 (1.90)	63.00 (1.93)	67.88 (7.54)	
Heavy	28.22 (1.74)	28.51 (1.78)	22.37 (6.76)	
MQI, kg/kg, mean ± SE	3.34 ± 0.02	3.35 ± 0.02	3.12 ± 0.11	.0338
MQI, % (SE)				.0048
Normal	51.67 (1.44)	52.54 (1.43)	33.84 (5.09)	
Low	18.56 (0.88)	18.35 (0.90)	22.91 (5.43)	
Extremely low	29.77 (1.51)	29.11 (1.48)	43.25 (5.40)	

CKM = cardiovascular-kidney-metabolic, MQI = muscle quality index, SE = standard error.

### 3.2. The relationship between MQI and advanced CKM syndrome

The association between MQI and advanced CKM syndrome is presented in Table 2. Both crude and adjusted logistic regression models demonstrated significant inverse associations, with higher MQI scores associated with reduced prevalence of advanced CKM syndrome. After full adjustment, each unit higher MQI was associated with 46% lower odds of advanced CKM syndrome (OR = 0.54, 95% CI = 0.29–0.99, *P* = .048). In categorical analysis, participants with extremely low MQI had significantly increased prevalence of advanced CKM syndrome compared with those having normal MQI levels in the fully adjusted model (OR = 2.21, 95% CI = 1.24–3.96, *P* = .011), with a significant linear trend observed across MQI categories (*P* for trend = .0091).

**Table 2:** Association of MQI with advanced CKM syndrome, weighted.

Variables	Model 1		Model 2		Model 3	
	OR (95% CI)	*P* value	OR (95% CI)	*P* value	OR (95% CI)	*P* value
MQI (continuous)	0.53 (0.31–0.90)	.026	0.56 (0.31–1.03)	.076	0.54 (0.29–0.99)	.048
Categories						
Normal	1 (reference)		1 (reference)		1 (reference)	
Low	1.94 (0.99–3.79)	.063	1.77 (0.89–3.50)	.115	1.86 (0.91–3.80)	.080
Extremely low	2.31 (1.47–3.61)	.001	2.17 (1.31–3.60)	.006	2.21 (1.24–3.96)	.011
*P* for trend	.0006		.0050		.0091	

Model 1: no covariates were adjusted.

Model 2: adjusted for sex, age, and race.

Model 3: adjusted for sex, age, race, education level, marital status, family income, smoking, and alcohol status.

CI = confidence interval, CKM = cardiovascular-kidney-metabolic, MQI = muscle quality index, OR = odds ratio.

### 3.3. A nonlinear relationship between MQI and advanced CKM syndrome

**Figure 2.**
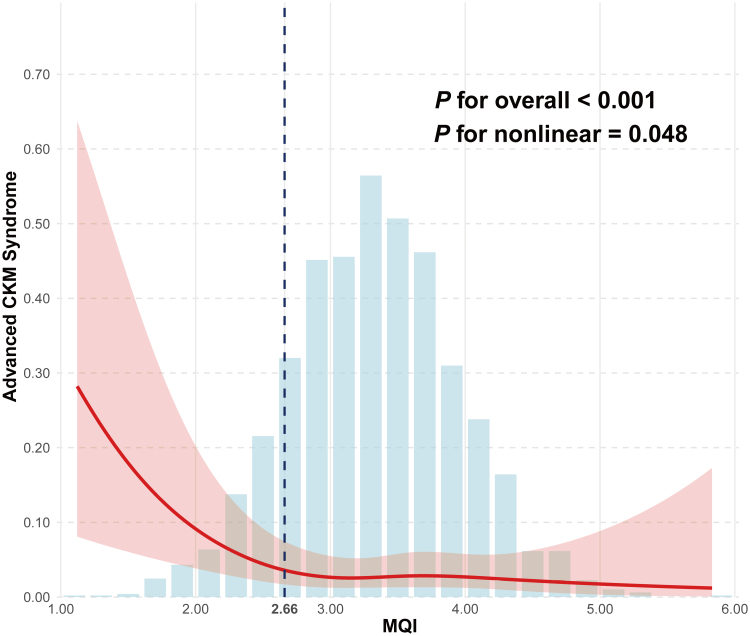
The dose–response relationship between MQI and advanced CKM syndrome was modeled using restricted cubic splines (overall association: *P* < .001; nonlinearity test: *P* = .048). Two-piecewise linear regression revealed a threshold effect with an optimal breakpoint at MQI = 2.66, indicating that the protective association of MQI with advanced CKM syndrome was most pronounced below this threshold and plateaued above it. CKM = cardiovascular-kidney-metabolic, MQI = muscle quality index.

Restricted cubic spline analysis demonstrated a significant nonlinear relationship between MQI and advanced CKM syndrome (nonlinearity test: *P* = .048, overall association: *P* < .001), depicted in Figure 2.

To further quantify this nonlinear relationship, we employed 2-piecewise linear regression to identify the threshold effect. As shown in Table 3, an optimal breakpoint was detected at MQI = 2.66 (*P* for log-likelihood ratio test = .005). Below this threshold, each unit increase in MQI was associated with an 86% lower prevalence of advanced CKM syndrome (OR = 0.14, 95% CI = 0.05–0.39, *P* = .0001), indicating a strong protective association. However, beyond the MQI value of 2.66, this association was substantially attenuated and became nonsignificant (OR = 0.92, 95% CI = 0.58–1.45, *P* = .7088).

**Table 3:** Threshold effect analysis of MQI on advanced CKM syndrome using a 2-piecewise linear regression model.

Variable	OR	95% CI	*P* value
Overall association	0.58	0.40–0.84	.0035
<2.66	0.14	0.05–0.39	.0001
>2.66	0.92	0.58–1.45	.7088
*P* for log-likelihood ratio test			.005

Adjusted for sex, age, race, education level, marital status, family income, smoking, and alcohol status.

CI = confidence interval, CKM = cardiovascular-kidney-metabolic, MQI = muscle quality index, OR = odds ratio.

### 3.4. Subgroup analysis and interaction

**Figure 3.**
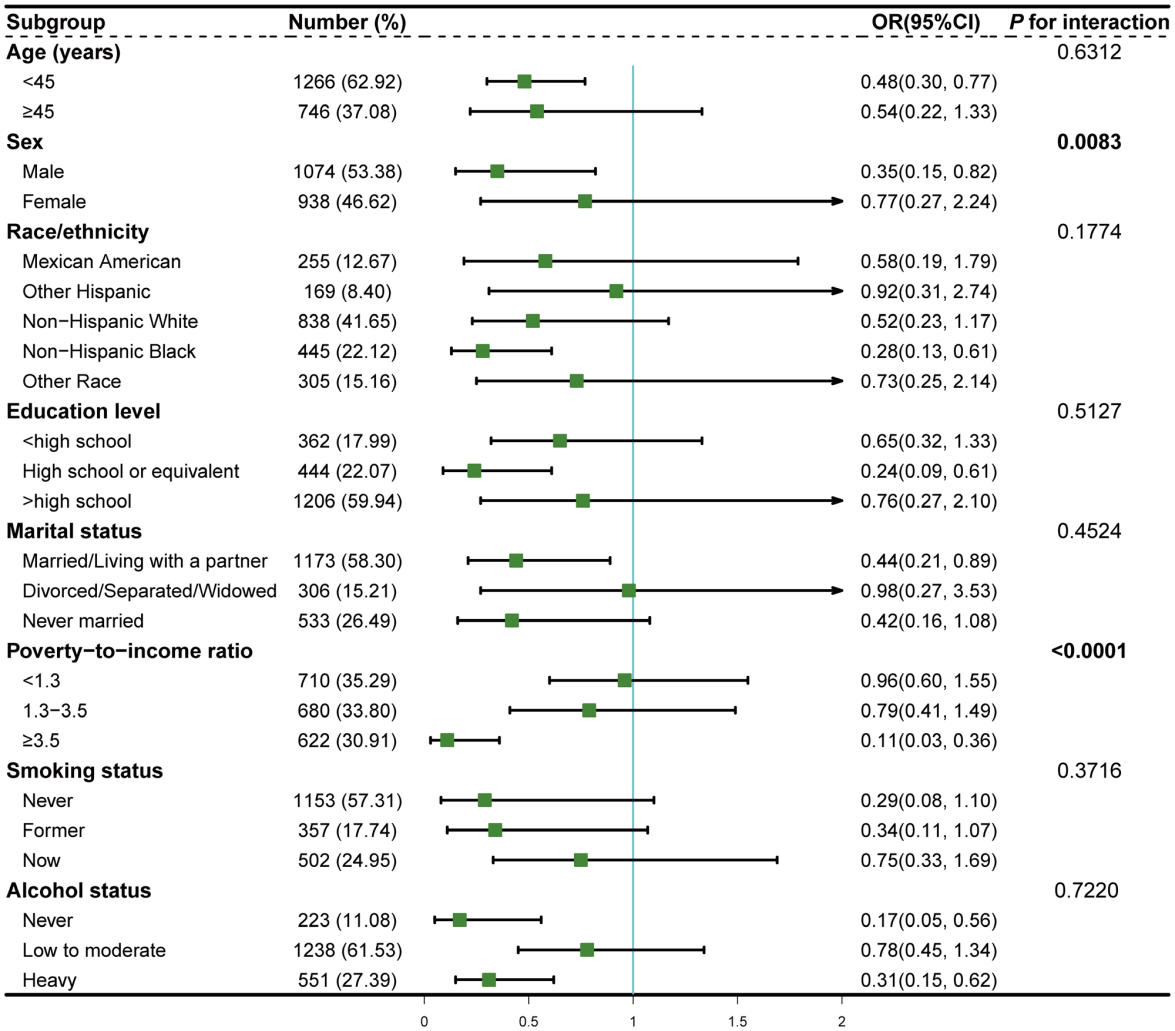
Forest plot of subgroup analyses examining the associations between MQI and advanced CKM syndrome. CI = confidence interval, CKM = cardiovascular-kidney-metabolic, MQI = muscle quality index, OR = odds ratio.

**Figure 4.**
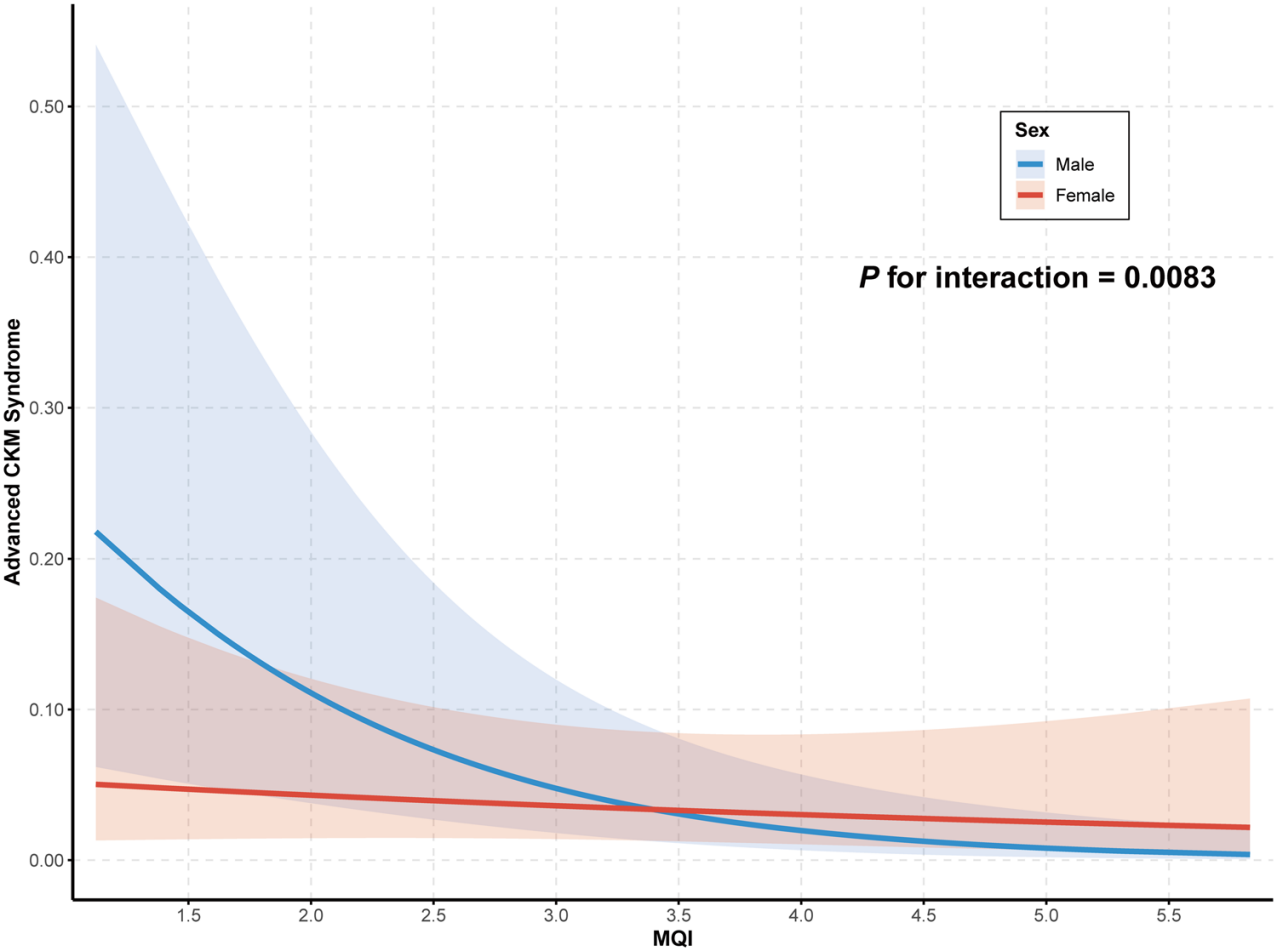
Association between MQI and advanced CKM syndrome by sex. The protective inverse association was statistically significant in males but not in females (*P* for interaction = .0083), suggesting that the protective effect of muscle quality differs by sex. CKM = cardiovascular-kidney-metabolic, MQI = muscle quality index.

**Figure 5.**
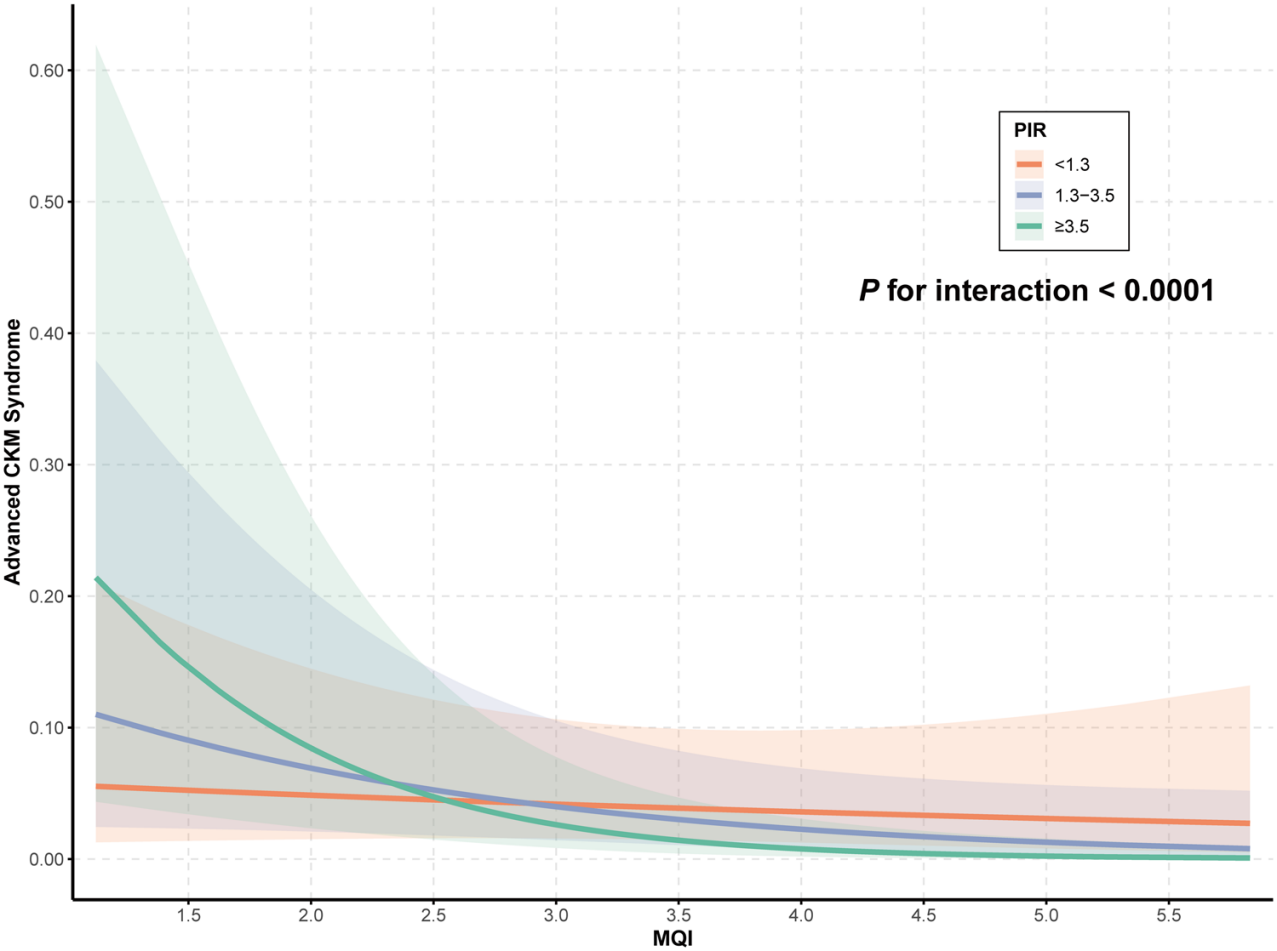
Association between MQI and advanced CKM syndrome across poverty-to-income ratio categories. The protective inverse association was statistically significant only in the highest-income group (≥3.5) but not in the lower income groups (<1.3 and 1.3–3.5; *P* for interaction < .0001), suggesting income level modifies the protective effect of muscle quality. CKM = cardiovascular-kidney-metabolic, MQI = muscle quality index.

Subgroup analyses revealed significant effect modifications by sex (*P* for interaction = .0083) and PIR (*P* for interaction < .0001), as detailed in the forest plot (Fig. 3). Specifically, data from Figure 3 showed that the inverse association between MQI and advanced CKM syndrome was statistically significant in males (OR = 0.35, 95% CI = 0.15–0.82) but not in females (OR = 0.77, 95% CI = 0.27–2.24), as visually represented in Figure 4. Similarly, for PIR, the association was significant in the highest category (≥3.5: OR = 0.11, 95% CI = 0.03–0.36) but not in the lower categories (<1.3: OR = 0.96, 95% CI = 0.60–1.55; 1.3–3.5: OR = 0.79, 95% CI = 0.41–1.49), as illustrated in Figure 5. No significant effect modifications were observed for the other stratifying factors (all *P* for interaction > .05).

Statistically significant inverse associations were also observed in several subgroups, including adults aged <45 years, non-Hispanic Black participants, those with high school or equivalent education, married/living with partner individuals, and both never-drinkers and heavy alcohol consumers.

## 4. Discussion

Our analysis provides novel evidence that higher MQI scores are associated with a lower prevalence of advanced CKM syndrome. A significant dose–response relationship was observed, wherein higher MQI levels correlate with reduced odds of the syndrome. Furthermore, we identified a significant nonlinear relationship with a critical threshold at MQI = 2.66, below which the protective association was particularly strong. Subgroup analyses revealed that these protective associations are more pronounced in males and high-income individuals. These findings provide novel epidemiological evidence supporting an inverse association between muscle quality and advanced CKM syndrome.

Our findings are consistent with emerging evidence demonstrating that muscle health confers benefits to cardiovascular, metabolic, and renal function. Meta-analyses indicate that higher HGS, which constitutes the numerator in MQI, is associated with a reduced risk of cardiovascular and all-cause mortality, both in general populations and among individuals with established CVD.^[5,6,31,32]^ Emerging evidence also shows that sarcopenia and low muscle mass are independent risk factors for CVD, metabolic disturbances, and adverse outcomes in CKD patients.^[8,33–36]^ By evaluating MQI as a ratio of muscle strength to skeletal muscle mass, our study extends previous findings and highlights the clinical relevance of muscle quality in the context of the comprehensive CKM syndrome.

Several biological mechanisms may explain our findings. Muscle tissue regulates glucose homeostasis and insulin sensitivity: impaired muscle quality contributes to metabolic deterioration and increased cardiometabolic risk.^[37,38]^ At lower MQI levels, reduced muscle quality may lead to metabolic dysfunction via decreased myokine secretion^[39]^ and the presence of sarcopenic obesity, which promotes chronic systemic inflammation.^[40]^ These interrelated mechanisms can increase susceptibility to the clustered risk factors that define CKM syndrome.

The observed nonlinear association with a threshold at MQI = 2.66 suggests a potential ceiling effect, where further improvements in muscle quality beyond this value offer limited incremental benefits. This plateau may be due to saturation of major metabolic and inflammatory pathways, such that the key pathogenic risks are already controlled once sufficient muscle quality is achieved. Therefore, enhancing muscle quality may yield the greatest clinical benefit among individuals with low baseline MQI.

Stratified analyses by sex revealed that the protective association between higher MQI and advanced CKM syndrome was more pronounced in male participants compared with female participants. This sex difference may be explained by the more rapid age-related decline in muscle mass and strength observed in men, resulting in lower muscular reserve and greater vulnerability to adverse outcomes.^[41,42]^ Men typically have higher absolute muscle mass compared to women,^[43]^ which may contribute to differences in insulin sensitivity and glucose metabolism between the sexes. Reduced muscle quality impairs insulin sensitivity^[44]^ and exacerbates renal dysfunction.^[45]^

In addition to the observed sex-specific effect, the protective association was also modified by socioeconomic status, as measured by the PIR. The stronger association between MQI and advanced CKM syndrome in high-income individuals is likely explained by several interconnected mechanisms. Higher-income groups typically have better nutritional status, including greater intake of quality protein and essential nutrients, supporting both muscle health and metabolic regulation.^[46,47]^ In addition, they are more likely to engage in regular physical activity and maintain healthier lifestyles, leading to improved muscle function and reduced cardiometabolic risk.^[48]^ Greater access to preventive healthcare and early intervention further facilitates the management of risk factors in this population.^[49]^ In contrast, individuals with lower income may face barriers such as limited access to nutritious foods, healthcare, and facilities for physical activity, which may attenuate the protective effects of higher MQI. These combined factors may account for the more pronounced benefits of higher MQI among high-income individuals.

Our analysis has several strengths. It is the first to examine nonlinear associations between MQI and advanced CKM syndrome in a large, diverse, and nationally representative sample. The use of robust statistical analyses, including nonlinear modeling and subgroup analyses, strengthens the credibility of our results. However, several limitations warrant consideration. First, the cross-sectional design of the NHANES study precludes causal inference and limits our ability to determine the exact directionality between MQI and CKM progression. Second, a substantial proportion of the initial NHANES sample was excluded due to missing data on MQI, CKM staging, or covariates, which may introduce potential selection bias. Third, CKM staging based on available clinical parameters may not comprehensively capture disease severity or progression. Fourth, despite rigorous covariate adjustment, residual confounding remains possible. Unmeasured factors such as physical activity intensity, dietary protein intake, and inflammatory markers could potentially influence the observed associations. Fifth, while it was methodologically necessary to exclude certain cardiometabolic variables from the adjustment models to avoid overadjustment, as these variables were incorporated into CKM staging, we acknowledge that this approach may leave some complex confounding pathways insufficiently addressed. Finally, future prospective longitudinal studies are warranted to validate these findings and clarify the potential causal relationship.

## 5. Conclusions

This study demonstrates a significant nonlinear association between MQI and advanced CKM syndrome, with a critical threshold at MQI = 2.66 and stronger protective effects in males and individuals of higher socioeconomic status. These findings highlight muscle quality as a potential factor associated with advanced CKM syndrome, suggesting that individuals with low MQI might benefit from clinical attention. Future prospective longitudinal studies are warranted to validate these observations.

## Author contributions

**Conceptualization:** Huang Yu.

**Formal analysis:** Huang Yu.

**Methodology:** Huang Yu.

**Visualization:** Huang Yu.

**Writing – original draft:** Huang Yu.

**Writing – review & editing:** Huang Yu.









## References

[1] Ndumele CE Neeland IJ Tuttle KR. A synopsis of the evidence for the science and clinical management of cardiovascular-kidney-metabolic (CKM) syndrome: a scientific statement from the American Heart Association. Circulation. 2023;148:1636–64. 37807920 10.1161/CIR.0000000000001186

[2] Aggarwal R Ostrominski JW Vaduganathan M. Prevalence of cardiovascular-kidney-metabolic syndrome stages in US adults, 2011-2020. JAMA. 2024;331:1858–60. 38717747 10.1001/jama.2024.6892 PMC11079779

[3] Minhas AMK Mathew RO Sperling LS. Prevalence of the cardiovascular-kidney-metabolic syndrome in the United States. J Am Coll Cardiol. 2024;83:1824–6. 38583160 10.1016/j.jacc.2024.03.368

[4] Wu Y Wang W Liu T Zhang D. Association of grip strength with risk of all-cause mortality, cardiovascular diseases, and cancer in community-dwelling populations: a meta-analysis of prospective cohort studies. J Am Med Dir Assoc. 2017;18:551.e517–35. 10.1016/j.jamda.2017.03.011 28549705

[5] López-Bueno R Andersen LL Koyanagi A. Thresholds of handgrip strength for all-cause, cancer, and cardiovascular mortality: a systematic review with dose-response meta-analysis. Ageing Res Rev. 2022;82:101778. 36332759 10.1016/j.arr.2022.101778

[6] Wang Y Pu X Zhu Z Sun W Xue L Ye J. Handgrip strength and the prognosis of patients with heart failure: a meta-analysis. Clin Cardiol. 2023;46:1173–84. 37469187 10.1002/clc.24063 PMC10577571

[7] Gao K Cao LF Ma WZ. Association between sarcopenia and cardiovascular disease among middle-aged and older adults: findings from the China Health and Retirement Longitudinal Study. EClinicalMedicine. 2022;44:101264. 35059617 10.1016/j.eclinm.2021.101264 PMC8760427

[8] Damluji AA Alfaraidhy M AlHajri N. Sarcopenia and cardiovascular diseases. Circulation. 2023;147:1534–53. 37186680 10.1161/CIRCULATIONAHA.123.064071 PMC10180053

[9] Wen Y Liu T Ma C. Association between handgrip strength and metabolic syndrome: a meta-analysis and systematic review. Front Nutr. 2022;9:996645. 36532558 10.3389/fnut.2022.996645 PMC9751936

[10] Jung HW Lee J Kim J. Handgrip strength is associated with metabolic syndrome and insulin resistance in children and adolescents: analysis of Korea National Health and Nutrition Examination Survey 2014-2018. J Obes Metab Syndr. 2022;31:334–44. 36581591 10.7570/jomes22053 PMC9828701

[11] Wu H Liu M Chi VTQ. Handgrip strength is inversely associated with metabolic syndrome and its separate components in middle aged and older adults: a large-scale population-based study. Metabolism. 2019;93:61–7. 30690038 10.1016/j.metabol.2019.01.011

[12] Kim K Park SM. Association of muscle mass and fat mass with insulin resistance and the prevalence of metabolic syndrome in Korean adults: a cross-sectional study. Sci Rep. 2018;8:2703. 29426839 10.1038/s41598-018-21168-5 PMC5807388

[13] Seo YG Song HJ Song YR. Fat-to-muscle ratio as a predictor of insulin resistance and metabolic syndrome in Korean adults. J Cachexia Sarcopenia Muscle. 2020;11:710–25. 32030917 10.1002/jcsm.12548 PMC7296262

[14] Xu H Li X Adams H Kubena K Guo S. Etiology of metabolic syndrome and dietary intervention. Int J Mol Sci. 2018;20:128. 30602666 10.3390/ijms20010128 PMC6337367

[15] Hwang SH Lee DH Min J Jeon JY. Handgrip strength as a predictor of all-cause mortality in patients with chronic kidney disease undergoing dialysis: a meta-analysis of prospective cohort studies. J Ren Nutr. 2019;29:471–9. 30827839 10.1053/j.jrn.2019.01.002

[16] Chen H Zhang F Huang L Bai Y Zhong Y Li Y. Thresholds of handgrip strength for all-cause mortality in patients with chronic kidney disease: a secondary systematic review with dose-response meta-analysis. Ren Fail. 2024;46:2305855. 38247440 10.1080/0886022X.2024.2305855 PMC10810645

[17] Ribeiro HS Neri SGR Oliveira JS Bennett PN Viana JL Lima RM. Association between sarcopenia and clinical outcomes in chronic kidney disease patients: a systematic review and meta-analysis. Clin Nutr. 2022;41:1131–40. 35430544 10.1016/j.clnu.2022.03.025

[18] Jiang L Xu L Sun W Bian K Wang Y. Association between the coexistence of chronic kidney disease and sarcopenia with cardiovascular disease and mortality. Aging Clin Exp Res. 2025;37:92. 40095245 10.1007/s40520-025-03003-w PMC11913966

[19] Wang Z Wu M Shao X Yang Q. Muscle quality index is associated with depression among non-elderly US adults. BMC Psychiatry. 2024;24:672. 39390450 10.1186/s12888-024-06136-w PMC11468283

[20] Xu B Jiang M Wei Y Duan R Tong F. Sex differences in the association between sleep duration and muscle quality index in adults: a cross-sectional study from NHANES 2011-2014. PLoS One. 2024;19:e0306661. 39008488 10.1371/journal.pone.0306661 PMC11249210

[21] Wang C Xu H Wang L. Muscle quality index correlates with arthritis: a cross-sectional study from NHANES 2011-2014. Front Med (Lausanne). 2025;12:1573729. 40395232 10.3389/fmed.2025.1573729 PMC12089042

[22] Hu Y Xu H Xie S Chen C Lei X. Relationship between muscle quality index and urinary incontinence among U.S. population: evidence from NHANES 2011 to 2014. Front Endocrinol (Lausanne). 2025;16:1533617. 40265166 10.3389/fendo.2025.1533617 PMC12011557

[23] Zhang M Lin H Xu X. Muscle quality index is correlated with insulin resistance and type 2 diabetes mellitus: a cross-sectional population-based study. BMC Public Health. 2025;25:497. 39915803 10.1186/s12889-025-21734-3 PMC11804045

[24] Wen H Li X Tan N. Inverse association between uric acid levels and muscle quality index in adults: a cross-sectional analysis of NHANES 2011-2014. BMC Public Health. 2024;24:3109. 39529042 10.1186/s12889-024-20559-w PMC11552229

[25] Shao Y Wang Y Jiang X. Muscle quality index and hyperuricemia: adipose tissue as a mediator. Front Endocrinol (Lausanne). 2025;16:1562837. 40496566 10.3389/fendo.2025.1562837 PMC12148888

[26] Lopes LCC Vaz-Gonçalves L Schincaglia RM. Sex and population-specific cutoff values of muscle quality index: results from NHANES 2011-2014. Clin Nutr. 2022;41:1328–34. 35576845 10.1016/j.clnu.2022.04.026

[27] Weng L Xu Z Chen Y Chen C. Associations between the muscle quality index and adult lung functions from NHANES 2011-2012. Front Public Health. 2023;11:1146456. 37234758 10.3389/fpubh.2023.1146456 PMC10206396

[28] Wen Z Gu J Chen R. Handgrip strength and muscle quality: results from the National Health and Nutrition Examination Survey database. J Clin Med. 2023;12:3184. 37176623 10.3390/jcm12093184 PMC10179381

[29] Ndumele CE Rangaswami J Chow SL. Cardiovascular-kidney-metabolic health: a presidential advisory from the American Heart Association. Circulation. 2023;148:1606–35. 37807924 10.1161/CIR.0000000000001184

[30] Tu D Sun J Wang P Xu Q Ma C. Overall sleep quality is associated with advanced stages in patients with cardiovascular-kidney-metabolic syndrome. J Am Heart Assoc. 2025;14:e038674. 40130386 10.1161/JAHA.124.038674 PMC12132850

[31] Xiao M Lu Y Li H Zhao Z. Association between handgrip strength and mortality of patients with coronary artery disease: a meta-analysis. Clin Cardiol. 2024;47:e24322. 39051437 10.1002/clc.24322 PMC11270052

[32] Soysal P Hurst C Demurtas J. Handgrip strength and health outcomes: umbrella review of systematic reviews with meta-analyses of observational studies. J Sport Health Sci. 2021;10:290–5. 32565244 10.1016/j.jshs.2020.06.009 PMC8167328

[33] Kim D Lee J Park R Oh C-M Moon S. Association of low muscle mass and obesity with increased all-cause and cardiovascular disease mortality in US adults. J Cachexia Sarcopenia Muscle. 2024;15:240–54. 38111085 10.1002/jcsm.13397 PMC10834318

[34] Li J Tu H Zhang Y Yang S Yu P Liu J. Risks of all-cause mortality in adults with chronic kidney disease with sarcopenia or obesity: a population-based study. J Cachexia Sarcopenia Muscle. 2025;16:e13828. 40468984 10.1002/jcsm.13828 PMC12138276

[35] Choi JW Kong SH Kim YJ. Effect of low muscle mass on total mortality related to metabolic disease in chronic kidney disease patients. Sci Rep. 2024;14:22837. 39354032 10.1038/s41598-024-73903-w PMC11445479

[36] Westbury LD Beaudart C Bruyère O. Recent sarcopenia definitions-prevalence, agreement and mortality associations among men: findings from population-based cohorts. J Cachexia Sarcopenia Muscle. 2023;14:565–75. 36604970 10.1002/jcsm.13160 PMC9891989

[37] DeFronzo RA Tripathy D. Skeletal muscle insulin resistance is the primary defect in type 2 diabetes. Diabetes Care. 2009;32:S157–163. 19875544 10.2337/dc09-S302 PMC2811436

[38] Cruz-Jentoft AJ Sayer AA. Sarcopenia. Lancet. 2019;393:2636–46. 31171417 10.1016/S0140-6736(19)31138-9

[39] Pedersen BK. Muscle as a secretory organ. Compr Physiol. 2013;3:1337–62. 23897689 10.1002/cphy.c120033

[40] Batsis JA Villareal DT. Sarcopenic obesity in older adults: aetiology, epidemiology and treatment strategies. Nat Rev Endocrinol. 2018;14:513–37. 30065268 10.1038/s41574-018-0062-9 PMC6241236

[41] Lowe R Hey P Sinclair M. The sex-specific prognostic utility of sarcopenia in cirrhosis. J Cachexia Sarcopenia Muscle. 2022;13:2608–15. 35945660 10.1002/jcsm.13059 PMC9745556

[42] Seino S Kitamura A Abe T. Dose-response relationships of sarcopenia parameters with incident disability and mortality in older Japanese adults. J Cachexia Sarcopenia Muscle. 2022;13:932–44. 35212170 10.1002/jcsm.12958 PMC8977959

[43] Janssen I Heymsfield SB Wang ZM Ross R. Skeletal muscle mass and distribution in 468 men and women aged 18-88 yr. J Appl Physiol (1985). 2000;89:81–8. 10904038 10.1152/jappl.2000.89.1.81

[44] Larsson L Degens H Li M. Sarcopenia: aging-related loss of muscle mass and function. Physiol Rev. 2019;99:427–511. 30427277 10.1152/physrev.00061.2017 PMC6442923

[45] Wang XH Mitch WE. Mechanisms of muscle wasting in chronic kidney disease. Nat Rev Nephrol. 2014;10:504–16. 24981816 10.1038/nrneph.2014.112 PMC4269363

[46] Paddon-Jones D Rasmussen BB. Dietary protein recommendations and the prevention of sarcopenia. Curr Opin Clin Nutr Metab Care. 2009;12:86–90. 19057193 10.1097/MCO.0b013e32831cef8b PMC2760315

[47] Bauer J Biolo G Cederholm T. Evidence-based recommendations for optimal dietary protein intake in older people: a position paper from the PROT-AGE Study Group. J Am Med Dir Assoc. 2013;14:542–59. 23867520 10.1016/j.jamda.2013.05.021

[48] Bauman AE Reis RS Sallis JF Wells JC Loos RJF Martin BW. Correlates of physical activity: why are some people physically active and others not? Lancet. 2012;380:258–71. 22818938 10.1016/S0140-6736(12)60735-1

[49] Adler NE Newman K. Socioeconomic disparities in health: pathways and policies. Health Aff (Millwood). 2002;21:60–76. 11900187 10.1377/hlthaff.21.2.60

