# Supplementary material for: Muscle quality index is associated with advanced stages in patients with cardiovascular-kidney-metabolic syndrome: A cross-sectional study
Source: Medicine (Baltimore). 2026 Jun 19;105(25):e49366. doi: 10.1097/MD.0000000000049366 (PMC13286380; doi:10.1097/MD.0000000000049366)
Supplement: Supplementary file 1 [file medi-105-e49366-s001.docx]

**Table S1** Sex-specific Cutoff value for MQI.

| Level | MQI (kg/kg) |
| --- | --- |
| Male |  |
| Normal > 1 SD | > 2.80 |
| 1 SD ≥ Low > 2 SD | 2.20-2.80 |
| Extremely low≤ 2 SD | ≤2.20 |
| Female |  |
| Normal > 1 SD | > 2.59 |
| 1 SD ≥ Low > 2 SD | 1.96-2.59 |
| Extremely low≤ 2 SD | ≤1.96 |

MQI = muscle quality index, SD = standard deviation.
