# Supplementary material for: Muscle quality index is associated with advanced stages in patients with cardiovascular-kidney-metabolic syndrome: A cross-sectional study
Source: Medicine (Baltimore). 2026 Jun 19;105(25):e49366. doi: 10.1097/MD.0000000000049366 (PMC13286380; doi:10.1097/MD.0000000000049366)
Supplement: Supplementary file 2 [file medi-105-e49366-s002.docx]

**Table S2 Cardiovascular-Kidney-Metabolic (CKM) Syndrome Staging Criteria.**

| Stage | Definition |
| --- | --- |
| Stage 0 | All of the following conditions must be met:   - Normal BMI (<25 kg/m² or <23 kg/m² if Asian ethnicity); - Normal waist circumference (<88/102 cm in women/men or <80/90 cm in women/men if Asian ethnicity); - Normoglycemia; - Normotension; - Normal lipid status; - No evidence of CKD; - No evidence of CVD |
| Stage 1 | Any of the following conditions:   - Elevated BMI (≥25 kg/m² or ≥23 kg/m² if Asian ethnicity); OR - Elevated waist circumference (≥88/102 cm in women/men or ≥80/90 cm in women/men if Asian ethnicity); OR - Prediabetes (fasting blood glucose 100-126 mg/dL or HbA1c 5.7-6.4%) |
| Stage 2 | Any of the following conditions:   - Elevated fasting serum triglycerides (≥135 mg/dL); OR - Hypertension (blood pressure ≥140/90 mmHg, self-reported history of hypertension, or current antihypertensive treatment); OR - Diabetes (fasting blood glucose ≥126 mg/dL, HbA1c ≥6.5%, self-reported history of diabetes, or current hypoglycemic treatment); OR - Metabolic syndrome (≥3 of the following: elevated waist circumference, HDL cholesterol <40 mg/dL for men and <50 mg/dL for women, fasting serum triglycerides ≥150 mg/dL, blood pressure ≥130/80 mmHg and/or current antihypertensive treatment, fasting blood glucose ≥100 mg/dL); OR - Moderate-to-high-risk CKD^1^ |
| Stage 3 | Any of the following conditions:   - Very-high-risk CKD^1^; OR - High predicted 10-year CVD risk^2^ |
| Stage 4 | Self-reported established CVD (coronary heart disease, angina, heart attack, heart failure, and stroke) |

^1^CKD risk categories are defined based on estimated glomerular filtration rate (eGFR) and urinary albumin-to-creatinine ratio according to established guidelines.

^2^The 10-year CVD risk was calculated using the Framingham risk calculation based on the Framingham risk score.

BMI = body mass index, CKD = chronic kidney disease, CKM = cardiovascular-kidney-metabolic, CVD = cardiovascular disease, eGFR = estimated glomerular filtration rate, HbA1c = glycated hemoglobin, HDL = high-density lipoprotein.
