# Supplementary material for: Muscle quality index is associated with advanced stages in patients with cardiovascular-kidney-metabolic syndrome: A cross-sectional study
Source: Medicine (Baltimore). 2026 Jun 19;105(25):e49366. doi: 10.1097/MD.0000000000049366 (PMC13286380; doi:10.1097/MD.0000000000049366)
Supplement: Supplementary file 3 [file medi-105-e49366-s003.docx]

**Table S3 Definitions and Classifications of Covariates.**

| Variable | Classification | Definition/Measurement |
| --- | --- | --- |
| Age | <45, ≥45 | Self-reported age in years |
| Sex | Male, Female | Self-reported sex |
| Race/ethnicity | Mexican American, Other Hispanic, Non-Hispanic White, Non-Hispanic Black, Other Race | Self-reported race/ethnicity categorized according to NHANES analytic guidelines |
| Education level | <High school, High school or equivalent, >High school | Self-reported highest level of education completed |
| Marital status | Married/Living with partner, Divorced/Separated/Widowed, Never married | Self-reported current marital status |
| Poverty-to-income ratio | <1.3, 1.3-3.5, ≥3.5 | Ratio of family income to poverty threshold, calculated by dividing family income by the poverty guidelines specific to family size, year, and state ^1,2^ |
| Smoking status | Never, Former, Current | Never: smoked <100 cigarettes in lifetime, Former: smoked more than 100 cigarettes in life and smoke not at all now, Current: smoked more than 100 cigarettes in life and smoke some days or every day |
| Alcohol status | Never, Low to moderate, Heavy | Never: had <12 drinks in lifetime, Low to moderate: had ≥12 drinks in lifetime but does not meet criteria for heavy drinking (<3 drinks per day for females, <4 drinks per day for males, and binge drinking on <5 days per month), Heavy:  ≥3 drinks per day for females, ≥4 drinks per day for males, or binge drinking (≥4 drinks on same occasion for females, ≥5 drinks on same occasion for males) on 5 or more days per month ^3^ |

NHANES = National Health and Nutrition Examination Survey.

**References:**

**1.** Johnson CL, Paulose-Ram R, Ogden CL, Carroll MD, Kruszon-Moran D, Dohrmann SM, Curtin LR. National health and nutrition examination survey: analytic guidelines, 1999-2010. *Vital Health Stat 2.* Sep 2013(161):1-24.

**2.** Ogden CL, Carroll MD, Fakhouri TH, Hales CM, Fryar CD, Li X, Freedman DS. Prevalence of Obesity Among Youths by Household Income and Education Level of Head of Household - United States 2011-2014. *MMWR Morb Mortal Wkly Rep.* Feb 16 2018;67(6):186-189.

**3.** Rattan P, Penrice DD, Ahn JC, et al. Inverse Association of Telomere Length With Liver Disease and Mortality in the US Population. *Hepatol Commun.* Feb 2022;6(2):399-410.
