# Supplementary material for: Muscle quality index is associated with advanced stages in patients with cardiovascular-kidney-metabolic syndrome: A cross-sectional study
Source: Medicine (Baltimore). 2026 Jun 19;105(25):e49366. doi: 10.1097/MD.0000000000049366 (PMC13286380; doi:10.1097/MD.0000000000049366)
Supplement: Supplementary file 4 [file medi-105-e49366-s004.docx]

**Table S4 Collinearity Assessment of Potential Covariates Using Variance Inflation Factor (VIF).**

| Variable | VIF |
| --- | --- |
| MQI | 1.1 |
| Sex | 1.1 |
| Age | 1.2 |
| Race/ethnicity | 1.1 |
| Education level | 1.3 |
| Marital status | 1.1 |
| Poverty-to-income ratio | 1.3 |
| Smoking status | 1.2 |
| Alcohol status | 1.2 |

VIF values < 5 indicate absence of substantial multicollinearity among the covariates.

MQI = muscle quality index, VIF = variance inflation factor.
